# What happens to our representation of identity as familiar faces age? Evidence from priming and identity aftereffects

**DOI:** 10.1111/bjop.12560

**Published:** 2022-03-11

**Authors:** Sarah Laurence, Kristen A. Baker, Valentina M. Proietti, Catherine J. Mondloch

**Affiliations:** ^1^ School of Psychology & Counselling Open University Milton Keynes UK; ^2^ Department of Psychology Brock University Canada University St. Catharines Ontario Canada; ^3^ Department of Psychology Trinity Western University Langley British Columbia Canada

**Keywords:** adaptation, face perception, facial ageing, familiarity, mental representation, priming

## Abstract

Matching identity in images of unfamiliar faces is error prone, but we can easily recognize highly variable images of familiar faces – even images taken decades apart. Recent theoretical development based on computational modelling can account for how we recognize extremely variable instances of the same identity. We provide complementary behavioural data by examining older adults’ representation of older celebrities who were also famous when young. In Experiment 1, participants completed a long‐lag repetition priming task in which primes and test stimuli were the same age or different ages. In Experiment 2, participants completed an identity after effects task in which the adapting stimulus was an older or young photograph of one celebrity and the test stimulus was a morph between the adapting identity and a different celebrity; the adapting stimulus was the same age as the test stimulus on some trials (e.g., both old) or a different age (e.g., adapter young, test stimulus old). The magnitude of priming and identity after effects were not influenced by whether the prime and adapting stimulus were the same age or different age as the test face. Collectively, our findings suggest that humans have one common mental representation for a familiar face (e.g., Paul McCartney) that incorporates visual changes across decades, rather than multiple age‐specific representations. These findings make novel predictions for state‐of‐the‐art algorithms (e.g., Deep Convolutional Neural Networks).

**Practitioner points**

- Our visual representations of familiar faces tolerate substantial changes in facial age.
- Behavioural data from priming and adaptation suggest age‐independent visual representations of familiar faces.

## INTRODUCTION

Two images of the same person can look very different. Faces change from moment to moment (e.g., lighting, expression) and from day to day (e.g., makeup, hairstyle, facial hair, health). Within‐person variability in appearance, coupled with similarity between identities (e.g., Matt Damon and Leonardo DiCaprio), makes unfamiliar face recognition prone to error; it is difficult to decide whether two photos belong to the same person or two different people (e.g., Bindemann & Sandford, 2011; Bruce et al., 1999; Hancock et al., 2000; Jenkins et al., 2011; Megreya & Burton, 2006; White et al., 2014). That same task is trivial when faces are familiar. Typically developing adults (and children over the age of six; Laurence & Mondloch, 2016) can recognize familiar faces across many different photographs (e.g., Bruce, 1982; Bruce et al., 2001; Johnston & Edmonds, 2009; Ritchie et al., 2015) – including those that capture real world experience with a face (e.g., the first 20 images from Google Images [Jenkins et al.] or Facebook [Laurence et al., 2016]). Recognizing facial identity requires perceivers to both discriminate a face from all other identities (*tell faces apart*) and recognize it despite variability in appearance (*tell faces together*). The striking difference in our ability to recognize unfamiliar versus familiar faces (reviewed in Andrews et al., 2015; Baker et al., 2017; Burton, 2013; Dowsett et al., 2016; Ritchie & Burton, 2017; Young & Burton, 2017, 2018). The nature of our mental representation of familiar faces remains unclear. To address this gap in the literature, we probed older adults’ representation of celebrities who were old at the time of our study and famous when much younger (i.e., who had aged alongside our participants).

Facial ageing poses a real challenge to face recognition because of the significant structural and textural changes that occur (see Albert et al., 2007; Deffenbacher et al., 1998; Kaur et al., 2015; Rhodes, 2009). A Google Image search for Paul McCartney neatly demonstrates how ageing changes our appearance substantially over many years. Paul McCartney, when he was in the Beatles, looks very different to his present appearance. Whereas matching identity in unfamiliar faces is especially challenging when the age gap between two images increases (Davis & Valentine, 2009; Megreya et al., 2013; Meissner et al., 2013), age‐related changes to appearance do not impair recognition of familiar faces. For example, in the Yearbook Task, participants were remarkably accurate in their ability to match photographs of their former classmates from their high school yearbook to images taken 24 to 26 years later (Bruck et al., 1991). Understanding the representations that support this ability will enrich models of recognition. We begin by reviewing key models that provide the context for our experiments.

### Models of face recognition

Valentine’s (1991) influential multi‐dimensional face space (MDFS) model emphasized discrimination (i.e., telling faces apart); each face is represented as a point in MDFS, the location of which is determined by how (e.g., larger vs. smaller eyes) and how much the face differs from a prototype. The MDFS model accounts for a range of phenomena (e.g., distinctiveness, caricature, inversion, and the own‐race advantage [Valentine]; age‐related change in childhood [Short et al., 2014]), but ignores familiarity and within‐person variability in appearance (see O’Toole et al., 2018). Nonetheless, adaptation aftereffects – an experimental paradigm used to explore how face images are organized in MDFS (Leopold et al., 2001; Little et al., 2005; Webster & MacLeod, 2011) provides numerous insights into how faces might be represented (e.g., the extent to which perception of identity is independent of changes in facial expressions (Fox et al., 2008; Mian & Mondloch, 2012) and (in this study) age.

Bruce and Young (1986) Young and Bruce influential model (2011) accounts for both familiarity and within‐person variability. Their model proposed that our visual representations of familiar faces are contained in *Face Recognition Units (FRUs)*. FRUs are activated by any recognizable image of a particular identity and, in turn, activate the associated *Person Identity Node (PIN)*. PINs are modality free (e.g., can be activated by non‐visual cues) and when they reach a certain threshold of activation, a person is recognized. FRUs are a useful concept, but the nature of these abstractive representations remains ill‐defined and a topic of recent debate.

One candidate is an average representation of each identity. Averages include visual information that is diagnostic of identity (i.e., consistent across images) and filter out non‐diagnostic information (see Burton et al., 2005). Several lines of evidence support the hypothesis that each familiar face is represented by an identity‐specific prototype. Models based on Principal Component Analyses (PCAs) of shape‐standardized images more accurately categorize new images of familiar (trained) identities when trained on averages as compared to when trained on multiple instances. Likewise, human participants have faster response times when making identity decisions based on face averages as compared to individual instances (Burton et al., 2005). Further evidence comes from ensemble coding. Adults and children automatically form an average after briefly viewing four different images of the same identity; they *recognize* the average when asked if it had been present in a study array (Davis et al., 2021; Kramer et al., 2015; Matthews et al., 2018).

An alternative candidate is a representation that stores variability in each identity's appearance – perhaps in addition to an average representation. Research published while the current manuscript was under review suggests a hybrid model whereby familiar(ized) faces are represented by a few age‐specific prototypes (e.g., young adult, middle‐aged adult, older adult) that might be linked semantically (Schneider & Carbon, 2021). The authors argue that an *exhaustive* prototype (i.e., a single prototype representing a face's appearance across the lifespan) is insufficient; as the time span over which the face must be represented increases, any single prototype decreases in utility. Evidence from ensemble coding also supports the hypothesis that humans store a representation of within‐person variability. Participants retain little information about individual exemplars when tested with low‐level object categories (i.e., they recognize the average but not the exemplars comprising that average; Ariely, 2001), but recognize both the average *and* individual exemplars when asked whether a particular face image had been in the study array (Kramer et al., 2015; Matthews et al., 2018; Neumann et al., 2013). The ability to retain a representation of exemplars while forming an average likely allows for a representation of familiar faces that includes both an average and a representation of idiosyncratic variability in appearance.

State‐of‐the‐art computer models also suggest that representations of identity might include a representation of variability. Models based on PCA accurately categorize new images of trained identities even when trained on instances that incorporate natural variability in appearance – if Linear Discriminant Analysis (LDA) is applied. LDA mimics top‐down processing; it increases distances between identities and groups different images of the same identity together (Kramer et al., 2017, 2018). Like PCA+LDA models, Deep Convolutional Neural Networks (DCNN) models suggest that representations of familiar identities include idiosyncratic variability in appearance. Whereas PCA‐based models require inputting shape‐normalized images (removing some between‐image variability), DCNNs are trained with raw images; nonetheless, DCNN models recognize newly learned identities accurately – even when disguised (Blauch et al., 2021; Noyes et al., 2021; O’Toole et al., 2018). DCNNs do not simply store an average representation of each identity. Rather, the identity code (the highest level of the model) for each image includes information about the image itself; as a result, a representation of variability is retained. Two lines of evidence show that DCNNs retain a representation of within‐identity variability. First, visualization of the top layer of face space shows that images of an identity are clustered based on viewpoint, lighting, expression, and whether the input was a still image or video (Colón et al., 2021; Hill et al., 2019). Second, classification of image attributes (e.g., expression, viewpoint, whether the input was a still image or video) based on output at the top levels of DCNNs is highly accurate (Colón et al.; Parde et al., 2017; see also Dhar et al., 2020 for *expressivity* as a measure of which image attributes are retained). Thus, whereas averages are posited to retain only information that is diagnostic of identity, both computer algorithms and data from humans suggest that familiar faces might be represented by individual instances rather than, or in addition to, an average.

Both PCA and DCNN models provide a promising avenue for theoretical understanding of our representations of faces, accounting for recognition despite age‐related change in appearance. Averages retain aspects of appearance that remain constant (Burton et al., 2005), although multiple age‐based averages might better represent identity (Schneider & Carbon, 2021). PCA+LDA models suggest that there is enough physical commonality between images to support recognition of familiar faces across their lifespan, suggesting that multiple different representations are not necessary (Mileva et al., 2020). Likewise, DCNNs store diverse images of the same face (e.g., images that vary in viewpoint, lighting, expression) in the same region of face space (Colón et al., 2021; Hill et al., 2019; see O’Toole et al., 2018 for a summary), suggesting that images from multiple decades might reside in close proximity. Nevertheless, any theory derived from computational models should be complemented by behavioural data from humans.

Own‐age biases in face recognition (e.g., Rhodes & Anastasi, 2012) suggest that faces of different ages might be represented separately. Human encounters with faces are linked to specific episodes, with recent images of familiar faces being recognized faster than past images (Kurth et al., 2015; Schneider & Carbon, 2021). Such findings suggest that humans may have age‐ (or episode‐) dependent representations. Unlike changes that occur from moment to moment (e.g., lighting, expressions, viewpoint), changes that arise from the ageing process are unidirectional, long term, and result from slow and progressive change. It is plausible that there might be a need to maintain multiple representations of a familiar face.

### The current study

This work aims to determine, using behavioural data from humans, whether we store a single mental representation of a familiar face that incorporates substantial age‐related changes (i.e., a single mental representation incorporating past and present appearance) or multiple age‐specific representations. This important question was highlighted as a direction for future research by Young and Bruce (2011): ‘Equally neglected is the question of what happens to our representations of familiar faces as they age? … Do we have one FRU [*Face recognition unit*; *visual representation*] for Paul McCartney when he was in the Beatles and one for when he was 64, or what? Why not several for the comparatively frequent changes in appearance during the Beatle years?’ (Young & Bruce, 2011, p. 970).

To the best of our knowledge, very little research has addressed this question and no study has collected behavioural data for familiar faces known to individuals for many years. Unlike computational models which are trained on multiple images of an identity taken at different ages simultaneously, older adults have seen faces age slowly, in real time. We applied two well‐established methodologies that have been used to examine our representations of faces and in a variety of other domains: long‐lag repetition priming (Experiment 1) and face adaptation aftereffects (Experiment 2). Our aim was not to differentiate whether the mental representation is average‐ versus Instance‐based; rather, we investigated whether older adults possess a single versus multiple mental representations of a highly familiar face.

Repetition priming occurs when prior exposure to a stimulus speeds up its subsequent recognition (e.g., Paul McCartney will be recognized faster if he was seen previously in an experiment; e.g., Bruce & Valentine, 1985; Calder & Young, 1996). Most previous research has examined repetition priming in young adults, but repetition priming is also observed in older adults (e.g., Pfütze et al., 2002; Wiese et al., 2017). Priming is greatest when the prime and target are identical images (though it is not affected by stretching – see Bindemann et al., 2008); the effect decreases as the similarity between the prime and test face decreases (Ellis et al., 1987, 1993; Johnston & Barry, 2001) and it is moderated by facial familiarity (Stevenage & Spreadbury, 2006). The neural underpinning of these effects is reflected in the event‐related brain potential (ERP) N250r, an ERP found for repetitions of different images of the same familiar (but not unfamiliar) face (Schweinberger et al., 1995). Repetition priming has been useful in informing models of face recognition (see Burton et al., 2011 for a discussion) making it a good candidate for examining our representations of familiar faces. Here, we examine whether long‐lag repetition priming is impacted by whether the age of the prime and target stimuli is the same or different.

Face adaptation aftereffects occur when prolonged exposure to a face alters perception of subsequent faces. Adaptation aftereffects have been observed for many different facial attributes (e.g., age, sex, race, identity, and expression; see Strobach & Carbon, 2013; Webster & MacLeod, 2011 for reviews). For example, after adaptation to older faces, subsequently presented faces appear younger (Schweinberger et al., 2010; O’Neil & Webster, 2011; O'Neil et al., 2014). Adaptation has been used to study representations of identity by asking participants to categorize ambiguous morphs (e.g., Paul McCartney and Steve Martin; see Fox et al., 2008; Hole, 2011; Mian & Mondloch, 2012). Adaptation to one identity (e.g., Paul McCartney) biases perception away from the adapted identity, such that ambiguous morphs are subsequently more likely to be judged as resembling the unadapted identity (e.g., Steve Martin). Such studies have shown that we have representations of facial identity that are invariant to changes in facial expression (Fox et al., 2008; Mian & Mondloch, 2012), stretching (Hole, 2011), and viewpoint (Hole, 2011; Jiang et al., 2007). As with priming, these techniques have proved useful for examining our representations of faces (see Rhodes, 2017). Here, we examine whether identity aftereffects are impacted by whether the age of the adapting and test face is the same or different.

If we have a single representation of a face that incorporates changes with age, then the magnitude of both repetition priming and identity aftereffects should be independent of whether the prime and adapting stimulus are the same age or different age as the test face. If we have separable age‐specific representations, then both repetition priming and identity aftereffects should be reduced when the prime or adapting stimulus is one age (e.g., young) and the test face is another (e.g., old). In this study, we presented images of celebrities when they were older (>60 years) and young (20 to 40 years). Evidence of age‐specific representations (e.g., stronger priming effects for same‐ vs. different‐age primes) would suggest at least two separable mental representations (FRUs), leaving open the question of how many. Because a model in which there is only a single representation of each identity is supported by the absence of significant differences, we followed up non‐significant difference in our frequentist analyses with Bayesian statistics to determine the strength of evidence in support of the null hypothesis.

## EXPERIMENT 1

In Experiment 1, we used long‐lag repetition priming to investigate how we represent the faces of people we have known for many years. Long‐lag priming has two phases: a priming phase in which a series of different identities are presented and a subsequent test phase, separated by a break. In the test phase, participants must indicate whether each face in a series is famous or unfamiliar. Some, but not all, of the famous faces in the test phase are also shown (or primed) in the priming phase. In this study, all test images showed celebrities when they were older (e.g., in their 70’s) and three types of primes were presented: identical images to those presented in the test phase, different images of the celebrities at the same age (e.g., Paul McCartney in his 70s), and images of the celebrities when they were younger (e.g., Susan Sarandon in her 30s). We compared response times for unprimed versus primed celebrities and examined the efficacy of same‐image, same‐age, and different‐age primes. Because long‐lag priming is influenced by similarity (Ellis et al., 1987; Johnston & Barry, 2001), similar priming effects for same‐ and different ‐age primes would provide strong support for the hypothesis that humans possess a single mental representation of familiar faces.

**FIGURE 1:**
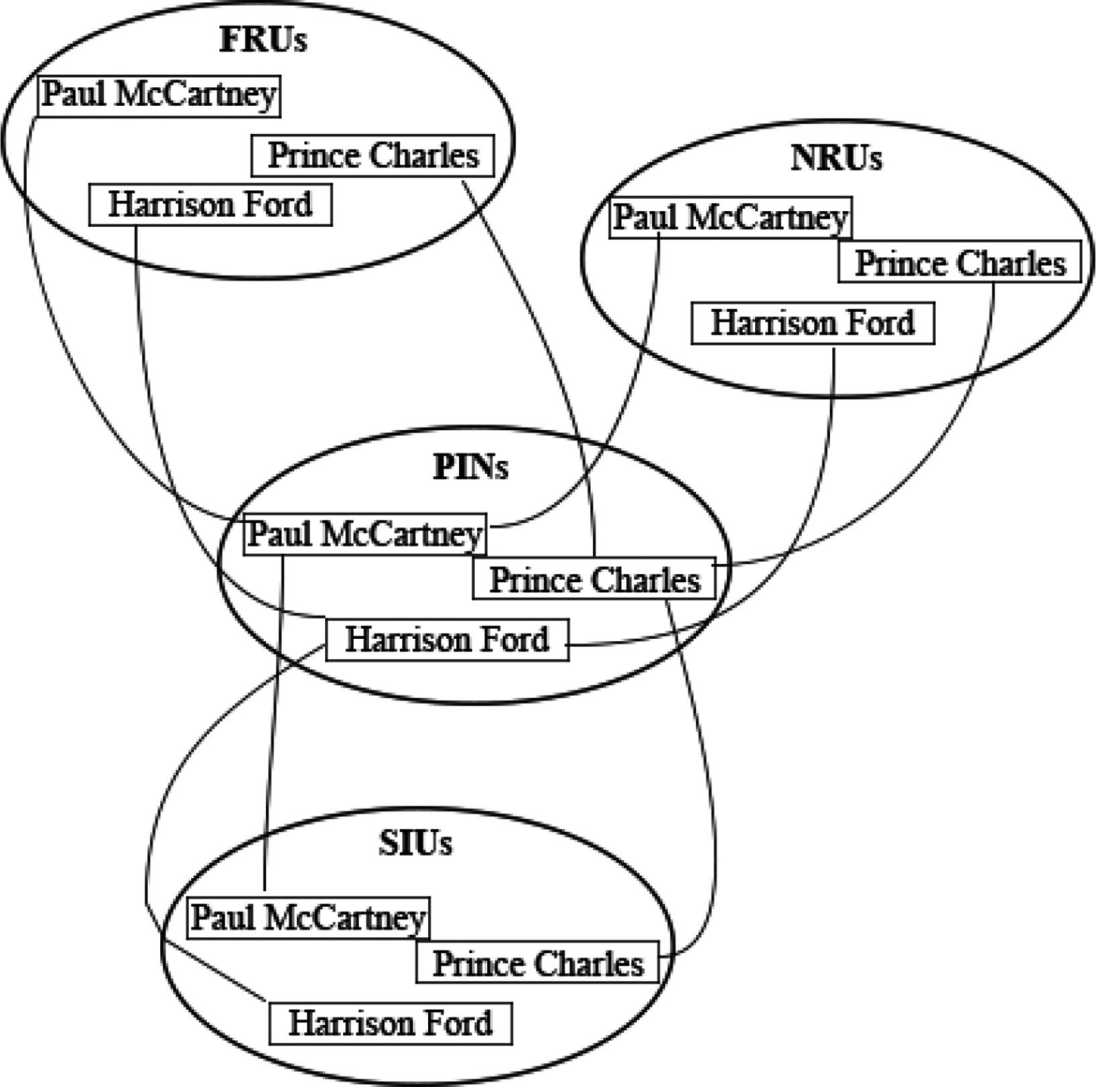
The IAC model of face recognition (Burton et al., 1990)

Our decision to use long‐lag priming was informed by Burton et al.’s (1990) IAC model of face processing. The IAC model proposes that there are pools of Face Recognition Units (FRUs), Person Identity Nodes (PINs), Name Recognition Units (NRUs), and Semantic Information Units (SIUs; see Figure 1). Units in different pools associated with the same person are connected via excitatory links and units belonging to different identities within a pool are connected by inhibitory links. Repetition priming for Paul McCartney occurs when exposure to Paul McCartney's face activates his FRU and activation spreads to his PIN. This spreading activation strengthens the link between the FRU and PIN. On a subsequent presentation of his face, activation will spread from the FRU to the PIN faster because of (a) the temporarily strengthened connection and (b) transient activation at the PIN; consequently, his face will be recognized faster. Long‐lag priming rules out facilitation attributable to transient activation at the PIN; inhibitory connections within pools result in intervening faces presented during the priming face (e.g., seeing Dustin Hoffman after Paul McCartney) inhibiting Paul McCartney's FRU and PIN. Only the strengthened link between the FRU and PIN remains intact. Thus, long‐lag priming can be used to determine whether photos from different ages activate the same or separate FRUs (i.e., whether adults possess a single vs. multiple mental representations of a highly familiar face). If there are separate FRUs for older and younger versions of the same identity (i.e., if images showing a celebrity when they are young vs. older activate different FRUs), RTs will be shorter for the same‐age (i.e., older) primes than the different‐age (i.e., younger) primes; the older FRU‐PIN link, but not the younger FRU‐PIN link, will be strengthened. If there is a single FRU that incorporates younger and older versions of the same identity (i.e., if images showing a celebrity when they are young vs. older activate a single FRU), then RTs will be comparable for same‐age and different‐age primes.

### Method

#### Participants

The final sample consisted of 43 participants (9 male) who were recruited to participate in the online study. This sample size provided more than enough power to detect a medium effect, as the minimum required participants was *N* = 35. Participants (*M*_age_ = 67.09, *SD* = 5.16) were blind to the purpose of the study. All participants confirmed they had normal or corrected‐to‐normal vision. Eight additional participants were tested, but were excluded as they recognized less than 3 identities for at least one of the priming conditions (*N* = 3 recognized fewer than three identities in only one condition, *N* = 2 recognized fewer than three identities in two conditions, *N* = 1 recognized fewer than thee identities in all three conditions) and two did not report having normal or corrected‐to‐normal vision.

#### Stimuli and design

The study followed a similar design to a study reported by Ellis et al. (1987, Experiment 3). We selected three images of 32 celebrities (half male, half female; e.g., Meryl Streep, Michael Cane). All of the chosen target celebrities were above 60 years of age and born between 1925 and 1958. Two images of each celebrity were recent photos (but taken at different events). The third image was an older photo of the respective identity, showing them at a younger age (around 30–40 years ago). Images were only selected if they were taken at a particular event that could be dated. All images, including fillers presented in test phase, were set to grayscale, cropped, and presented at 190 x 285 pixels.

One recent photo of each target celebrity was assigned to the test phase. As in Ellis et al. (1987), these were intermixed with 20 images of famous individuals that were novel for all participants (i.e., never primed) and 52 images of unfamiliar individuals (over the age of 60, half male/female); including images of novel celebrities ensures that most of the familiar faces were not previously seen in Phase 1. We selected images of German celebrities (not famous in Canada) for the unfamiliar filler items, in order to match the style of the celebrity images.

Images of the target celebrities were divided into four sets, such that eight celebrities (half male/female) were in each set. Each set comprised eight *same*‐*image* primes (i.e., the image presented in the test phase), eight *same*‐*age* primes (the alternate recent photo), and eight *different*‐*age* primes (the image of the celebrity from when they were younger). Thus each set included primes for 24 of the target celebrities, leaving eight celebrities unprimed. The condition to which each target identity was assigned was counterbalanced across sets (e.g., was unprimed for some participants and primed in each of the three conditions for others).

The distractor phase contained illusory‐face and object‐matched images that were used by Wardle et al. (2020). Each of the 64 images (*N* = 32 illusory faces) were set to colour, cropped, and presented at 310 x 310 pixels.

#### Procedure

Similar to Ellis et al.’s study (1987), the priming and test phase were presented as two different tasks to participants. Stimuli were presented in Testable software (www.testable.org). After an introduction, the priming phase started. Participants were presented 24 images (eight from each condition); images were presented in the centre of the screen for 9 seconds each. After the image display, participants were asked to provide a name and/or other identifying information. Participants provided as much or little information as they could via the keyboard, using the text entry tool. After pressing ENTER, the next image appeared on the screen after an 800 ms break. Participants were not told the identity of the celebrity when they were unable to identify them.

After the priming phase, participants completed a face‐detection task, which functioned as the distractor task. For each image, participants were asked to indicate if the object was face‐like (by pressing the ‘f’ key) or not face‐like (by pressing the ‘j’ key). Images were presented sequentially in the centre of the screen. To keep the duration of the distractor task comparable across participants, images were shown for 3 s, and then participants were provided with a 2 s time window to respond. Following their response and an 800 ms break‐interval, participants were shown the next image. On average, participants took 5.39 minutes (*SD* = 1.56) to complete the task.

Prior to beginning the test phase, participants completed four practice trials; two of which presented famous faces. The test phase comprised 104 test images that were presented individually in randomized order at the centre of the screen. Participants were instructed to decide for each image, whether the person they saw was famous (f key on the keyboard) or not famous (j key on the keyboard) as quickly and as accurately as possible.

### Results and discussion

Across each of the three conditions, participants recognized almost all of the eight identities in the priming phase (Same‐Image condition: *M* = 6.00, *SD* = 1.54; Same‐Age condition: *M*= 6.19, *SD* = 1.55; Different‐Age condition *M* = 6.05, *SD* = 1.5). For each participant, only the identities that were recognized in the priming phase were included in the analyses for the test phase. Errors in identifying the celebrities could occur in the priming phase or in the test phase by judging a famous face as not famous. As pointed out by Ellis et al. (1987), we have no way of knowing whether the latter error was due to a failure to recognize the celebrity or due to an accidental incorrect response. Error rates in the test phase were rare (*same*‐*image* = 0.85%, *same*‐*age* = 2.62%, *different*‐*age* = 8.70%, *unprimed* = 15.99%) and were not analysed further.

The average response times to recognize target celebrity faces as famous for each of our four conditions are shown in Table 1. Mauchly's Test of Sphericity was violated (*p* = 0.03), thus Greenhouse–Geisser corrections were applied. A one‐way ANOVA revealed a significant main effect of condition (*F*(2.59, 108.94) = 20.29, *p* < 0.001, ηp^2^ = 0.33. Planned contrasts revealed that response times were faster for the three primed (*same*‐*image*, *same*‐*age*, *different*‐*age*) conditions than the *unprimed* condition (*F*(1,42) = 43.74, *p* <0.001). Reaction times were faster for the *same*‐*image* condition than the two different image conditions (*same*‐*age*, *different*‐*age*) (*F*(1,42) = 24.79, *p* < 0.001). There was no difference between reaction times for the *same*‐*age* condition and the *different*‐*age* priming condition (*F*(1,42) = 0.68, *p* = 0.41). To determine whether there was support for the null hypothesis (i.e., no difference in response times in the same‐ vs. different‐age condition), we followed up this non‐significant effect with a Bayesian t‐test in JASP (2019). We used the default prior in JASP (effect size Cohen's d, with a Cauchy distribution with scale r = 0.707) and found moderate support for the null hypothesis (BF_01_ = 4.40).

**Table 1:** Mean reaction time for each condition (milliseconds)

	*M*	*SD*
Same‐image	945.93	196.19
Same‐age	1116.62	275.35
Different‐age	1141.45	327.60
Unprimed	1248.83	309.99

Our findings support the hypothesis that a single representation can incorporate age‐related changes in appearance, consistent with evidence from computer models (Mileva et al., 2020). Using the conceptualization of the IAC model (Burton et al., 1990), there appears to be one FRU‐PIN link for each celebrity, regardless of age. From this, we can infer a single FRU for each identity as the use of long‐lag repetition priming allows us to rule out the PIN as the locus of these effects. Note that this need not have been the case. Our remarkable ability to recognize highly familiar faces despite the changes that occur with ageing, does not imply that they possess a single representation (FRU); just as familiarity judgements can be based on voices and name – each of which can activate a Person Identity Node, it is possible that multiple age‐specific FRUs for a single identity converge on a single PIN. Our findings from repetition priming suggest multiple FRU‐PIN links (and therefore multiple FRUs) is unlikely; however, prior to drawing strong conclusions, we tested a new group of older adults on a different paradigm: Adaptation aftereffects.

## EXPERIMENT 2

Like priming, adaptation has been used to examine our representations of identity. Research using this technique has been interpreted within the MDFS model of face processing (see Mueller et al., 2021; Strobach & Carbon, 2013 for reviews), which suggests that faces are encoded relative to a norm (i.e., the centre of face space; e.g., Leopold et al., 2001). Researchers have examined how faces are represented within face space by manipulating the similarity between the adaptation and test stimuli (e.g., in terms of size, contrast, orientation, viewpoint, expression; see Strobach & Carbon, 2013). The degree to which adaptation transfers from the adaptor to the test face can be used to infer the extent to which both stimuli are coded by shared neural populations (i.e., are located in the same region of face space). Identity aftereffects for familiar (Hole, 2011) and familiarized faces have been found to transfer across viewpoint to a greater extent than for less familiar faces (Jiang et al., 2007), suggesting that our representations of familiar identities in face space generalize across multiple different viewpoints (unlike our view‐specific representations of unfamiliar faces). Likewise, identity aftereffects for familiar (and unfamiliar) faces are independent of whether the adapting and test faces display the same or different facial expressions, suggesting that our representations of identity are expression invariant (Fox et al., 2008; Mian & Mondloch, 2012). In Experiment 2, a similar approach was taken to establish whether our representations of familiar faces generalize across multiple different ages (i.e., whether identity aftereffects for familiar faces are independent of whether the adapting and test faces are the same or different ages).

Older adults were adapted to two familiar celebrities (e.g., Paul McCartney and Steve Martin). The adapting faces were young on some trials (e.g., Paul McCartney when he was in the Beatles) and older in others (e.g., Paul McCartney in his 70s). Participants subsequently made identity decisions with morphed faces (e.g., 50% Paul McCartney/50% Steve Martin) that were young on some trials and older on others. On half the trials, the morph faces were the same age as the adapting faces (age congruent) and on the other half the morph faces were of a different age than the adapting faces (age incongruent). Based on the results of Experiment 1, we predicted comparable adaptation in both congruent and incongruent conditions. Such a finding would suggest that older and younger versions of the same identity have overlapping representations. This is because if old and young versions of the same identity have independent or separable representations (e.g., one representation for Paul McCartney from the present day and one for when he was in the Beatles), then aftereffects should be present in the age‐congruent conditions, but reduced in the age‐incongruent condition.

### METHOD

#### Participants

Thirty‐seven older adults living in independent housing in the Niagara region of Ontario, Canada (27 female; *M* = 72.68, *SD* = 5.13; Range = 61–83) participated. This sample was more than adequate to detect a medium‐sized effect, as the minimum number of participants would be *N* = 22. All participants confirmed they had normal or corrected‐to‐normal vision and were familiar with both young and old images of the celebrities we used in the experiment.

#### Materials

We selected 12 celebrities (8 males and 4 females) whose faces were used as stimuli; celebrities were born between 1935 and 1949. As with Experiment 1, we chose celebrities who were famous when they were both younger and older (i.e., they were famous when the experiment was carried out and when our older adult participants were young). We used these 12 celebrities to create six same‐sex pairs. Members of each pair were born between 3 and 12 years (*Median* = 3 years) apart. Two images of each celebrity were recent (from 2012/2013) and two were images from when the celebrity was younger (23 to 37 years of age). We determined age by selecting images taken at events that could be dated. The median age difference within younger image pairs (6 pairs x 2 views) was 5 years (*Range* = 1 to 14). For each time point, one of the images was a frontal view, and the other image was a roughly ¾ view (20**°** to 50**°** away from frontal view). Images were obtained using a Google image search and were chosen using the following criteria: the image showed a frontal or ¾ view of the face with a neutral expression, there was nothing covering the face (e.g., glasses) and the image was of a good quality. All images were converted to grayscale, resized so that the width of the head was 150 pixels, and cropped so that only the head was visible.

We paired up the 12 celebrities to create six same‐sex face pairs. For each face pair, we created two morph continua from the frontal‐view – one between the young images of the two celebrities and one between the older images of the two celebrities. For each continuum, morph software was used to make 13 morphs ranging between 20% Person A/80% Person B and 80% Person A/20% Person B in 5% increments. The morphs, and the 100% identity strength images from which the morphs were generated, were cropped to an oval containing just the internal features of the face. Morphed faces served as test faces and original ¾‐view faces served as adapting stimuli.

#### Procedure

Each participant was assigned to one face pair. The face pair to which they were assigned was determined by a pre‐screening procedure completed prior to the experiment proper. All participants were tested individually.

##### Pre screening

At the beginning of the testing session, participants completed a familiarity test in which they were presented with a name checklist of 12 celebrities; the names were presented in their pre‐assigned pairs. Participants were required to check off name pairs with whom they were familiar, and told that they should only indicate a name pair was familiar if they knew both identities.

Participants subsequently viewed two images each of the celebrities whose names were indicated as familiar: one younger image and one older image. We asked participants to rate how familiar they were with each celebrity, at the age they were in the picture, on a scale from 1 (highly unfamiliar) to 7 (highly familiar). This was to ensure that participants were familiar with old and young images of each celebrity; a face was only considered to be familiar to the participant if they recognized the young and old pictures of their face (indicated by a score of >4). Out of all the face pairs with which the participant was familiar, one pair was selected by the experimenter to be included in the experiment proper with the aim of balancing the number of participants tested with each identity pair as much as possible. Familiarity scores for selected pairs were comparable for the two members of each pair (M_diff_ = .25 on our 7‐point scale); the lowest familiarity score was 5 averaged across the two image time points. The images used in the familiarity check were the 100% identity strength, frontal view images that subsequently appeared in the first training block of the adaptation experiment; however, the images shown in this during pre‐screening were not cropped to an oval.

##### Adaptation experiment

Participants were seated approximately 60 cms from a computer screen. The following describes the procedure for participants assigned to the face pair Paul McCartney and Steve Martin. The procedure was exactly the same for all other face pairs, except the identities varied.

##### Training

Participants initially completed two training blocks during which only frontal‐view faces were presented; each block contained a total of eight trials. On‐screen instructions told participants that they would be presented with images of Paul McCartney and Steve Martin when they were old (in one block) and young (in another block) and that they should indicate whether each face belongs to Paul McCartney or Steve Martin by pressing the key that corresponded to the first letter of that person's name (e.g., ‘P’ for Paul McCartney or ‘S’ for Steve Martin). Participants viewed four 100% images of each identity in each block. Each face was presented for 300 ms, preceded by a fixation cross for 750 ms, and followed by a response screen prompting participants to respond. The response screen remained visible until a response was made. The order in which participants viewed the old/young block was counterbalanced, and the order of trials within each block was randomized for each participant.

Participants then completed a further two training blocks; each block comprised 13 trials. Participants were told that this time they would see ambiguous (blend) morphs between Paul McCartney and Steve Martin when they were old (in one block) and young (in the other block); they were told that some of the morphs might look more like one of the identities and others would be ambiguous. Participants were asked to indicate who they thought each morph looked more like, but that there were no right or wrong answers and they should answer on the basis of their first impression. In each block, participants viewed each of the 13 morphs between the two identities for 300 ms in a manner identical to the first two training blocks. As with the first two blocks, the order in which participants viewed the old/young block was counterbalanced, and the order of trials within each block was random.

##### Adaptation phase

Each participant completed four adaptation blocks, the order of which was counterbalanced across participants. In two blocks, the age of the adaptor was old and in two blocks the age of the adaptor was young. For each age of adaptor, the age of the test morph was congruent in one block (old adaptor/old morph; young adaptor/young morph) and incongruent in another block (old adaptor/ young morph; young adaptor/old morph).

At the start of each block, participants were told that they would see two faces on each trial. They were asked to attend carefully to the first face (a three‐quarter‐view face) presented on each trial and then indicated to which of the two identities (e.g., Paul McCartney or Steve Martin) the second face belonged. Each block included 26 trials. The trial format was similar to the method used by Fox et al. (2008). Each trial had the same sequence of events: A 750 ms fixation cross, a 5000 ms adapting face (Person A or B), a 1000 ms mask, a 300 ms morphed probe face, and a response screen. Within each block, each participant saw 13 trials in which they adapted to Person A and 13 trials in which they adapted to Person B.

An identity aftereffect was measured by computing a difference score. For each participant, we subtracted the number of trials on which they thought the morphed face resembled Person A after adapting to Person A from the number of trials on which they thought the morphed face resembled Person A after adapting to Person B. Positive difference scores indicate an identity aftereffect had occurred. An identity aftereffect was calculated for each block.

### Results and discussion

To determine whether aftereffects occurred in each testing condition, we conducted one‐sample t tests in each of the four conditions against zero (zero = no aftereffect); significant aftereffects were observed in all conditions [young–young: *t* (36) = 2.40, *p* = .02; young–old: *t* (36) = 2.74, *p* = .01; old–old: *t* (36) = 4.91, *p* <.001; old–young: *t* (36) = 4.14, *p* <.001] with small to medium effect size for the blocks with young adult adaptors (both congruent, Cohen's d_young‐young_ = 0.39 and incongruent, Cohen's d_young‐old_ = 0.45) and medium to large effect size for the blocks with older adult adaptors (both congruent, Cohen's d_old‐old_ = 0.81, and incongruent, Cohen's d_old‐young_ = 0.68). For the *M*s and *SD*s, see Table 2.

**Table 2:** Mean adaptation scores across all four conditions

Adaptation	*M*	*SD*
Young‐young	0.81	2.05
Young‐old	0.89	1.98
Old‐old	1.54	1.91
Old‐young	1.08	1.59

The size of the aftereffects was analysed using a repeated measures analysis of variance (ANOVA) with two factors: *age of the adapting face* (old vs. young) and *age of the test morph* (congruent vs. incongruent with adapting age). Aftereffects were comparable regardless of whether the adapting face was young or old [*F*(1, 36) = 2.06, *p* = .16, ηp^2^ = .05] and regardless of whether the age of the adapting face and test morph were congruent or incongruent [*F*(1, 36) = .52, *p* = .47, ηp^2^ = .01]. The interaction was also non‐significant [*F*(1, 36) = .83, *p* = .37, ηp^2^ = .02]. Thus, despite finding evidence of adaptation aftereffects in every condition, we found no evidence that the magnitude of aftereffects varied with facial age or the congruency of facial age between adapting and test stimuli.

As with Experiment 1, we also conducted Bayesian analyses to follow up on our null result for *age of the test morph* using the default options and priors in JASP. Consistent with the frequentist analysis, the Bayesian analysis revealed moderate evidence for the null hypothesis (i.e., no difference between the congruent vs. incongruent condition; BF_01_ = 4.17. This pattern complements the frequentist analysis and suggests our results are more likely under the null hypothesis (i.e., there is no effect of congruency between the age of the adapting and test face on the size of the adaptation aftereffect). In short, consistent with the findings of Experiments 1, we found no evidence of separable representations of young versus older faces.

## GENERAL DISCUSSION

Young and Bruce (2011) raised the question of how we represent faces as they age:

‘Equally neglected is the question of what happens to our representations of familiar faces as they age?… Do we have one FRU for Paul McCartney when he was in the Beatles and one for when he was 64, or what?’ (Young & Bruce, 2011, p. 970). Contemporary models of face recognition suggest that a single representation may suffice (see Mileva et al., 2020; O’Toole et al., 2018), but no study to date had directly examined this question in human participants. We did so, using two complementary approaches. In each case we tested older adults, a population that had learned familiar faces over decades (i.e., as they aged).

In Experiment 1, we found equivalent repetition priming for same‐identity primes regardless of whether the primes were the same age as the target or a different age. In Experiment 2, we found comparable identity adaptation aftereffects regardless of whether the adaptor was the same age as the test face or a different age. The absence of any effect of facial age on priming and adaptation aftereffects was found in the context of significant priming and adaptation effects and confirmed by Bayesian analyses. Our face recognition mechanisms seem to cope with facial ageing, arguably an extreme form of within‐person variability, by updating a common representation – analogous to the FRUs in Bruce and Young (1986) Young and Bruce (2011) model. Whereas Schneider and Carbon (2021) suggest that episodic prototypes of familiar faces are linked semantically (e.g., via the PIN of Bruce & Young's model) our data suggest that individual exemplars and/or episodic prototypes of familiar faces comprise a single visual representation. This conclusion aligns with Burton et al. (2016) who argue that learning a new face involves ‘incorporating many superficially different stimuli into a common representation’ (pp. 218) and complements computational modelling by Mileva et al. (2020) who argue that images taken across the lifespan have enough physical similarity between them such that it is not necessary to store multiple different representations of an individual identity.

### The nature of our representations

Our study was not designed to determine whether recognition is achieved via face averaging (Burton et al., 2005; Valentine, 2001) or via storing exemplars – a model supported by DCNNs (Blauch et al., 2021; Longmore et al., 2008; O’Toole et al., 2018). Rather, our study was designed to test the extent to which a single representation can incorporate perceptual changes associated with ageing.

Our data complement PCA+LDA models of face recognition – which suggest that multiple different representations are not necessary for familiar face recognition (Mileva et al., 2020), and DCNN models – which suggest we store diverse images of a familiar face in the same region of face space (see O’Toole et al., 2018 for a summary). DCNNs are characterized by a hierarchical organization such that identity is nested within gender, and illumination and viewpoint are nested within identity (Hill et al., 2019; O’Toole et al., 2018). Our data extend these models by suggesting that identity is not nested within age – a model that would predict stronger priming and aftereffects when primes/adapters are the same age as test stimuli. This has intuitive appeal: Whereas gender is a stable characteristic, age is not – though age‐related changes, unlike changes in illumination and viewpoint, occur slowly.

Further research is needed to examine how humans and DCNNs represent familiar faces as they age. As noted by Schneider and Carbon (2021), ageing has been neglected in models of face recognition. Their proposed Episodic Prototype Model suggests that each identity is associated with small number of prototypes, each representing a different time period (e.g., young adult, middle age, older adult; Schneider & Carbon, 2021). We suggest that while our representations of highly familiar faces may be biased by more recent encounters with an identity, recognition of a face (e.g., an image of Paul McCartney) is achieved by activating the entire identity region (e.g., the Paul McCartney region). Future work should determine whether age is nested within identity for familiar faces known for many years – perhaps including a small number of prototypes, and reconcile this with recency effects (e.g., more recent encounters with a familiar face being more central to our representations).

Older adults provided an ideal opportunity to examine how age is accounted for in representations of familiar faces. The remarkable ability of humans to recognize thousands of faces (Jenkins et al., 2018) requires discriminating each face from other identities and recognizing it despite within‐person variability – a challenge that is enhanced by disguise (Noyes & Jenkins, 2019) and ageing. Within‐person variability in appearance is, in part, idiosyncratic (Burton et al., 2016; Kramer et al., 2018); thus, to some extent learning must be identity‐specific, although some learning generalizes across identities (see Blauch et al., 2021). Humans and computer models benefit from increased exposure to variability in a to‐be‐recognized face (Baker et al., 2017; Burton et al., 2016; Dowsett et al., 2016; Kramer et al., 2018; Mileva et al., 2020; Murphy et al., 2015; Noyes et al., 2021; Ritchie & Burton, 2017) and from exposure to how that face differs from others (Mundy, Honey, & Dwyer, 2007; Noyes et al., 2021). Top‐down processing also plays a key role; when labels are absent, randomly assigned, or conceptually unrelated performance is poor (Andrews et al., 2015; Cavazos et al., 2019; Kramer et al., 2018; Mileva et al., 2020; O’Toole et al., 2018; Schwartz & Yovel, 2016). Like well‐performing DCNN models, our older adult participants had a wealth of general face knowledge and abundant experience with the celebrities with whom they were tested (analogous to high quality of training data in DCNNs).

Three characteristics of our task conditions might have enhanced the use of a single age‐independent representation. First, the older adults in our study were contemporaries with the celebrities. They knew the celebrities when they were both young (e.g., when the Beatles first became famous) and aged alongside them. What we have shown is that if you have aged slowly with people over time you are able to incorporate age changes into a common representation. Perhaps this is because the age changes are gradual, allowing our representations to slowly update. The same might not be true of young adults, at least for familiar celebrity faces. Because they will have encountered both young and older instances of a celebrity simultaneously rather than seeing faces change slowly over time, young adults might store separable representations. Future research should determine how flexible our representations are of familiar faces under these learning conditions.

Second, we showed participants images of celebrities taken when they were a young versus older adult and all images were taken after the celebrity was famous. We did not present images of the celebrity from childhood – images that differ even more from current‐day appearance and may not be integrated into a representation of the celebrity as a singer/actor. It remains possible that images from childhood are not integrated into the same representation.

Third, we used celebrity faces as our familiar face stimuli. Previous research using repetition priming has found differences between personally familiar faces and celebrity faces (Herzmann et al., 2004; Keyes & Zalicks, 2016). The nature of our exposure to personally familiar faces likely differs from our exposure to celebrity faces (also see Wiese et al., 2019). We encounter celebrities at many different ages simultaneously; for example, a Google Images search for Paul McCartney returns images of him both old and young. In contrast, our exposure to personally familiar faces is heavily biased towards their current appearance. Consequently, our representations of personally familiar faces might be more biased by an identity's recent appearance than past appearance (e.g., the central tendency of our representations might be biased be more recent encounters). Our future research aims to determine the extent to which differential experience with celebrities versus personally familiar faces leads to differences in their representations. Nonetheless, our findings provide strong evidence that a representation of a familiar face can incorporate tremendous variability in appearance – allowing for accurate recognition across a wealth of instances while accurately discriminating that identity from others that are similar in appearance (see Burton et al., 2016; Jenkins et al., 2011).

### Insights about older adults

In addition to understanding what happens to mental representations for faces as they age, the current research examined how familiar faces are represented in healthy older adults. Past research has shown that ageing is associated with various deficits in unfamiliar face recognition (e.g., increased false alarms [Fulton & Bartlett, 1991]; lower accuracy in identifying faces from a line‐up [Searcy et al., 1999]; poorer matching of unfamiliar faces [Megreya & Bindemann, 2015]) and familiar face recognition (e.g., reduced N250 priming effects [Wiese et al., 2017]; poorer recognition of younger celebrity faces, but not older celebrity faces [Bartlett et al., 1991]). Our findings suggest older adults have robust representations of familiar faces that tolerate within‐person variability in ageing. Future studies should address the extent to which older adults are able to build a robust representation for newly encountered faces and compare the nature of representations for newly learned faces versus faces known for many years.

## CONCLUSION

Recent theoretical development based on computational models of face recognition suggest highly variable images of an individual may be integrated into a common representation. The aim of our studies was to provide complementary behavioural data to determine whether we incorporate age changes into a common representation, or whether the changes in appearance associated with ageing are so substantial that we represent a lifetime's experience with a familiar face as a collection of separable representations. Using two established behavioural techniques, our work suggests we incorporate substantial changes in appearance associated with age into a common representation – providing an answer to a long‐standing question posed by Bruce and Young and evidence in support of recent computer models.

## CONFLICT OF INTEREST

All authors declare no conflict of interest.

## AUTHOR CONTRIBUTION

**Sarah Laurence:** Conceptualization; Data curation; Formal analysis; Funding acquisition; Methodology; Resources; Software; Supervision; Validation; Writing – original draft; Writing – review & editing. **Kristen A. Baker:** Data curation; Formal analysis; Investigation; Methodology; Project administration; Resources; Software; Validation; Visualization; Writing – original draft; Writing – review & editing. **Valentina M. Proietti:** Formal analysis; Investigation; Methodology; Project administration; Supervision; Visualization; Writing – review & editing. **Catherine J. Mondloch:** Conceptualization; Funding acquisition; Formal analysis; Methodology; Resources; Supervision; Validation; Writing – original draft; Writing – review & editing.

## Data Availability

The data that support the findings of this study are openly available in the OSF at https://osf.io/c6nh3/?view_only=a0fce6c102b548f197196514be078c28.
